# Clinical characteristics and managements of severe hand, foot and mouth disease caused by enterovirus A71 and coxsackievirus A16 in Shanghai, China

**DOI:** 10.1186/s12879-019-3878-6

**Published:** 2019-03-27

**Authors:** Kang Cai, Yizhong Wang, Zhongqin Guo, Huiju Yu, Huajun Li, Liya Zhang, Shanshan Xu, Qingli Zhang

**Affiliations:** 1 Department of Pediatrics, Xinhua Hospital, Shanghai Jiao Tong University School of Medicine, 1665 Kongjiang Road, Shanghai, 200092 China; 2 Department of Infectious Diseases, Shanghai Children’s Hospital, Shanghai Jiao Tong University, 355 Luding Road, Shanghai, 200062 China; 3 School of Public Health and Management, Ningxia Medical University, Yinchuan, China; 4 Department of Pediatrics, Xinhua Hospital, Shanghai Jiao Tong University School of Medicine, Chongming Branch, Shanghai, China

**Keywords:** Hand, Foot and mouth disease, Enterovirus A71, Coxsackievirus A16, Intravenous gamma globulin intervention, Children

## Abstract

**Background:**

Hand, foot and mouth disease (HFMD) is a transmissible infectious disease caused by human enteroviruses (EV). Here, we described features of children with severe HFMD caused by EV-A71 or coxsackievirus A16 (CV-A16) in Shanghai, China.

**Methods:**

Severe EV-A71 or CV-A16 caused HFMD children admitted to the Xinhua Hospital from January 2014 and December 2016, were recruited retrospectively to the study. Symptoms and findings at the time of hospitalization, laboratory tests, treatments, length of stay and residual findings at discharge were systematically recorded and analyzed.

**Results:**

Of 19,995 children visited clinic service with probable HFMD, 574 children (2.87%) were admitted, 234 children (40.76%) were confirmed with EV-A71 (90/574) or CV-A16 (144/574) disease. Most (91.02%) of the patients were under 5 years. Initial clinical symptoms of EV-A71 and CV-A16 cases were: fever > 39 °C in 81 (90%) and 119 (82.63%), vomiting in 31 (34.44%) and 28 (19.44%), myoclonic twitching in 19 (21.11%) and 11(7.64%), startle in 21 (23.33%) and 20 (13.69%), respectively. Serum levels of interleukin-1β (IL-1β), IL-2, IL-6, IL-8, interferon-γ (IFN-γ), tumor necrosis factor-α (TNF-α) were significantly upregulated in severe HFMD subjects. Forty-seven children (20.08%) treated with intravenous gamma globulin (IVIG) showed decreased duration of illness episodes. All children were discharged without complications.

**Conclusions:**

EV-A71 and CV-A16 accounted 40.76% of admitted HFMD during 2014 to 2016 in Xinhua Hospital. IVIG appeared to be beneficial in shortening the duration of illness episodes of severe HFMD.

## Background

Hand, foot and mouth disease (HFMD) is a common childhood infectious disease caused by human enteroviruses (EV). HFMD is prevalent worldwide, particularly in the Asia-Pacific region, such as outbreaks in Singapore, Republic of Korea, Vietnam, Hong Kong, and mainland of China in past decades [1–5]. It has been showed that the outbreaks of HFMD occur year round and are associated with meteorological, environmental and socioeconomic factors [6]. Children under 5 years old are the high risk population of HFMD [7]. HFMD is usually a mild, self-limiting illness with typical clinical manifestations including fever, inappetence, erythrasma, vesiculation on hands and feet, and vesicles in the mouth [5]. Most of the HFMD cases recover spontaneously in a few days without complications. However, neurologic and systemic complications, such as encephalomyelitis, aseptic meningitis, acute flaccid paralysis, and even brainstem encephalitis, can developed rapidly in a minority of cases [7]. In critical severe HFMD cases, autonomic dysregulation, pulmonary oedema and myocardial impairment occur and can lead to death [7].

As the causative pathogen of HFMD, EV are a group of positive sense single-stranded RNA viruses belong to *Enterovirus* genus, *Picornaviridae* family [8]. EV are divided into four species (EV-A, EV-B, EV-C and EV-D) based on the viral genetic characteristics [8]. It was reported that most of HFMD cases were caused by species A, including EV-A71 and coxsackievirus A16 (CV-A16) [9]. In past decades HFMD outbreaks worldwide causing epidemics were reportedly due to EV-A71 and CV-A16 [10, 11]. In addition, other serotypes such as CV-A4, CV-A5, CV-A6, CV-B2, and CV-B3 were also identified in HFMD cases [7]. Recently, some of them are becoming more prevalent in some regions, for example, CV-A6 and CV-A10 were responsible for several outbreaks of HFMD in Asia, America and Europe since 2010 [12–14].

After several large HFMD outbreaks during 2007 and early 2008, HFMD was defined as a C-class notifiable communicable disease by Centers for Disease Control and Prevention (CDC) of China. A national surveillance system was established to monitor the epidemiology and aetiology of HFMD since 2008. The Chinese HFMD surveillance systems were mainly focused on EV-A71 and CV-A16 due to their predominance in China [15]. The severity of EV-A71 and CV-A16 ranges widely from mild to severe systemic damages. EV-A71 related severe neurologic diseases and fatal cases were previous reported in China [16, 17]. Severe and fatal cases of HFMD caused by CV-A16 were also reported [18]. Here, we conducted a retrospective study to analyze the clinical features, managements and outcomes of severe HFMD cases caused by EV-A71 or CV-A16 from 2014 to 2016 admitted in a tertiary care hospital of Shanghai, China.

## Methods

### Study cohort

Two hundred and thirty-four children diagnosed as severe HFMD were retrospective recruited to the study cohort from the Pediatric Infectious Department, Xinhua Hospital, Shanghai Jiao Tong University School of Medicine, China, from January 2014 and December 2016. Inclusion criteria were children aged 1 month to 14 years, with severe EV-A71 or CV-A16 HFMD which required hospital admission under the Pediatric Department of Infectious Diseases at Xinhua Hospital. A probable HFMD case was defined as a patient with papular-vesicular rash on hands, feet, mouth, or buttocks, with or without fever. A confirmed case was defined as a probable case with laboratory evidence of EV infection [5]. Mild HFMD was defined as oral ulcers, maculopapular or papular-vesicular rash on the hands, feet and buttock, accompanied with or without fever. Patients were classified as severe if they had any neurological complications (aseptic meningitis, encephalitis, encephalomyelitis, acute flaccid paralysis, or autonomic nervous system dysregulation), or cardiopulmonary complications (pulmonary edema, pulmonary hemorrhage, or cardiorespiratory failure), or both [5]. Children with significant underlying disease (5 children with congenital heart disease, 20 with iron deficiency anemia, and 2 with cerebral palsy), and children with mild EV-A71 or CVA16 HFMD who do not require admission were excluded from the study. Aseptic meningitis was defined as cerebral spinal fluid (CSF) protein > 450 mg/L and/or ≥ 40 white blood cells (WBC)/mm. The criteria for admission to the Pediatric Departments of Infectious Diseases included the following symptoms/signs: prolonged hyperthermia (axillary temperature > 39 °C for 2 days or more), very unwell general appearance and/or neurological findings, such as reduced level of consciousness, limb weakness, ataxia or seizures [19]. EV-A71 and CV-A16 infections were confirmed by real time RT-PCR (Shanghai Zhijiang Biotechnology Science and Technology Company, Shanghai, China) from stool, nasopharyngeal swab and/or CSF specimens of the subjects. Only patients with laboratory-proven EV-A71 or CV-A16 from 1 or more clinical specimens were enrolled in the study. Fifty mild EV-A71 and 50 CV-A16 HFMD outpatients, and 100 gender and age matched healthy individuals from children for health examination in Department of Children Healthcare, Xinhua Hospital were enrolled as control groups. Written informed consent was obtained from parents or legal guardians of children eligible for study enrollment. This study was approved by the Regional Ethical Review Board in Xinhua Hospital.

### Antiviral treatments

Hospitalized HFMD children were treated with ribavirin (RBV) or intravenous gamma globulin (IVIG) depended on the clinical symptoms. Indications for antiviral treatment: children with fever, rashes on hands, feet, mouth and buttocks (macular papules, papules, small herpes), and accompanied with cough, runny nose, loss of appetite were treated with ribavirin (RBV spray via oral cavity at 0.5 mg per dose, 3 times per day, for 7 days); children with prolonged hyperthermia (> 39 °C), and/or neurologic complications were treated with IVIG at 1 g/kg, given twice daily, for 2 days [19]. All patients were given ribavirin. However, only partial HFMD children with neurologic complications received IVIG in addition to the ribavirin because of the high price and safety concern of IVIG.

### Serum cytokine measurement

The serum cytokines levels of severe HFMD, interleukin-1β (IL-1β), IL-2, IL-6, IL-8, IL-10, interferon-γ (IFN-γ), tumor necrosis factor-α (TNF-α) were determined by using the IMMULITE-1000 Immunoassay System (Siemens Healthcare Global), an automated microbead-based analyzer that makes use of chemiluminescent technology for analyte detection, which was performed according to the manufacturer’s instructions [20]. Serum cytokines levels of 50 mild EV-A71, and 50 CV-A16 HFMD cases, and 100 healthy individuals were also measured.

### Data collection

The following data were systematically extracted from the hospital records of enrolled children: symptoms and findings at the time of hospitalization, laboratory test results, length of hospital stay, treatments and residual clinical findings at the time of discharge.

### Statistical analysis

Statistical analysis was performed between healthy control, mild and severe EV-A71 and CV-A16 HFMD groups. Different groups were compared for initial clinical manifestations, gender, age, length of febrile period, presence of seizure, length of stay, laboratory test results, cytokine levels, treatment with immunoglobulin and treatment outcome. Mann-Whitney U test was used to compare difference of two independent groups, and Kruskal-Wallis H test was used to compare the difference between multiple independent groups. All data are presented as median with interquartile range (IQR). All statistical analyses were performed with SPSS 24 for Windows. Statistical significance was defined as *P* < 0.05.

## Results

### Clinical and demographic characteristics of severe EV-A71 and CV-A16 HFMD

**Fig. 1:**
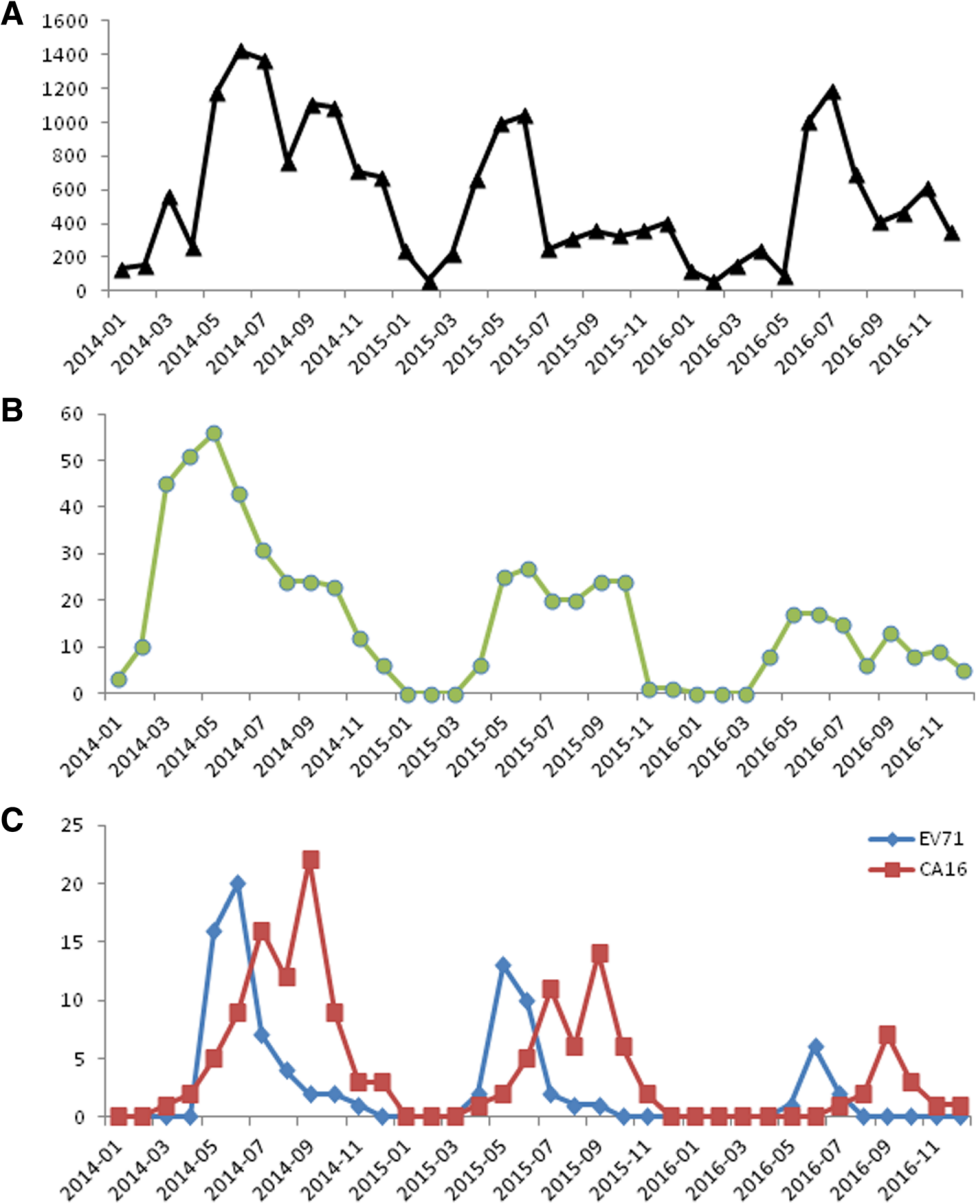
The seasonal pattern of HFMD clinic visits (**a**), admissions (**b**), and EV-A71, CV-A16 admissions (**c**) from January 2014 to December 2016 in Xinhua Hospital

From January 1, 2014 to December 31, 2016, 19,995 children visited Xinhua Hospital clinic service with probable HFMD with or without aseptic meningitis. Of these children, 574 children (574/19,995, 2.87%) were admitted to Xinhua Hospital after clinical assessment. Of those admitted subjects, 234 children (234/574, 40.76%) presented with EV-A71 (90/574, 15.68%) or CV-A16 (144/574, 25.08%) confirmed severe enough disease for enrollment in the study (Table 1). None of the children required pediatric intensive care unit (PICU) care. The HFMD seasonal pattern for clinic visits and hospitalizations were shown in Fig. 1A and B. Most admissions of EV-A71-related HFMD were occurred in May, June and July during the first epidemic peak (Fig. 1C). And majority of CV-A16 confirmed subjects were admitted in July, August and September during the second epidemic peak (Fig. 1C). As shown in Table 1, of the 234 eligible enrolled subjects, there were more males than females (M/F, 136:98), and the median age was 29 months (range: 4 months to 8 years) with 213 (213/234, 91.02%) children under 5 years (Table 1). Initial clinical symptoms of EV-A71 and CV-A16 confirmed children were: fever > 39 °C in 81 (81/90, 90%) and 119 (119/144, 82.63%), nausea in 3 (3/90, 3.33%) and 2 (2/144, 1.39%), vomiting in 31 (31/90, 34.44%) and 28 (28/144, 19.44%), myoclonic twitching in 19 (19/90, 21.11%) and 11 (11/144, 7.64%), startle in 21 (21/90, 23.33%) and 20 (20/144, 13.69%), respectively. The incidence of vomiting and myoclonic twitching symptoms were significantly higher in EV-A71 HFMD than CV-A16 HFMD (Table 1). Laboratory tests showed that white blood cell (WBC), C reactive protein (CRP), creatinekinase MB (CKMB) were elevated in most of hospitalized subjects. Blood sugar (BS) and alanine aminotransferase (ALT) were elevated in some of subjects. High WBC and total protein in CSF were detected in 19 (19/234, 8.11%) children (Table 1). The number of subjects with high WBC and total protein in CSF were significantly higher in EV-A71 HFMD than CV-16 HFMD (Table 1).

**Table 1:** Clinical and demographic characteristics of EV-A71 and CV-A16 caused severe HFMD

Variable	EV-A71 (90) n (%)	CV-A16 (144) n (%)	χ^2^	*P*
Age				
< 1	8	14	0.789	0.852
1–3	48	75		
3–5	23	45		
> 5	11	23		
Gender (M)	51(56.67)	85(59.02)	0.127	0.722
WBC (> 15 × 10^9^/L)	67(74.44)	79(54.86)	9.052	0.003
CRP (> 40 mg/L)	59(65.56)	86(59.72)	0.800	0.371
BS (> 6 .8mmol/L)	10(11.11)	9(6.25)	1.754	0.185
CKMB (> 25 IU/L)	63(70)	92(63.89)	0.925	0.336
ALT (> 40 IU/L)	9(10)	9(6.25)	1.097	0.295
WBC in CSF (> 40 × 10^6^/L)	10(11.11)	1(0.69)	13.415	0.000
Protein in CSF (> 450 mg/L)	9(10)	1(0.69)	11.724	0.001
Fever	81(90)	119(82.63)	2.417	0.12
Nausea	3(3.33)	2(1.39)	1.001	0.317
Vomiting	31(34.44)	28(19.44)	6.609	0.01
Myoclonic twitching	19(21.11)	11(7.64)	8.994	0.003
Startle	21(23.33)	20(13.89)	3.418	0.064
IVIG	28(31.11)	19(13.19)	11.076	0.001

Abbreviations: *WBC* white blood cell, *CRP* C reactive protein, *BS* blood sugar, *CKMB* creatinekinase MB, *ALT* alanine aminotransferase, *CSF* cerebral spinal fluid, *IVIG* intravenous gamma globulin intervention

### Serum cytokines levels of severe EV-A71 and CV-A16 HFMD

As shown in Table 2, there was no significant difference of cytokine levels between mild HFMD and healthy controls. We found that cytokine levels of EV-A71 and CV-A16 HFMD were significantly elevated compared with either mild HFMD or healthy control group. There was no significantly difference between EV-A71 and CV-A16 group in most of those cytokines. Subgroup analysis showed that cytokine levels of both EV-A71 and CV-A16 HFMD cases treated with or without IVIG were significantly higher than healthy controls (Tables 3, and 4). However, there were no statistical differences of cytokine levels between EV-A71 and CV-A16 HFMD cases treated with or without IVIG. The cytokines levels were significantly decreased after antiviral treatment with or without IVIG (Tables 3 and 4). In addition, there was no statistical difference of cytokine level between different age groups of HFMD subjects (data not shown).

**Table 2:** Serum cytokines levels between healthy control, mild HFMD, severe EV-A71, and CV-A16 HFMD before treatment

Cytokine (pg/mL)	Control (100) M (IQR)	Mild (100) M (IQR)	Severe EV-A71 (90) M (IQR)	Severe CV-A16 (144) M (IQR)	χ2	*P*
IL-1β	3.00(2.95,3.53)^a^	3.30(3.00,3.50)^a^	15.30(8.48,42.70)^b^	16.00(8.65,67.00)^b^	192.69	< 0.001
IL-2	441.00(350.25,543.25)^a^	460.00(356.75,574.00)^a^	1017.00(725.00,1472.00)^b^	1133.00(838.25,1500.75)^b^	142.29	< 0.001
IL-6	4.05(3.20,4.70)^a^	4.20(3.60,5.10) ^a^	16.00(6.21,84.90)^b^	12.65(5.31,69.23)^b^	143.68	< 0.001
IL-8	46.00(36.75,52.75)^a^	47.00(38.00,52.00)^a,b^	221.50(53.13,652.50)^c^	63.45(21.43,478.75)^a,d^	85.25	< 0.001
IL-10	5.10(3.68,6.25)^a^	5.50(3.90,6.48)^a^	8.01(6.30,12.10)^b^	8.31(5.92,14.70)^b^	49.38	< 0.001
IFN-γ	4.00(1.60,6.50)^a^	5.70(2.50,10.10) ^a^	44.50(37.75,52.00)^b^	58.40 (35.90,69.85)^b^	28.95	< 0.001
TNF-α	4.65(3.75,5.40)^a^	4.70(3.85,5.70)^a^	25.40(16.20,40.85)^b^	19.85 (13.93,50.88)^b^	146.90	< 0.001

Abbreviations: *M* median, *IQR* interquartile range, *IL* interleukin, *IFN*, interferon, *TNF* tumor necrosis factor

a vs b, b vs c, a vs c, statistical significance

**Table 3:** Comparison of serum cytokines levels of healthy control, severe EV-A71 HFMD before and after treated with IVIG + RBV, and severe EV-A71 HFMD before and after treated with RBV only

Cytokine (pg/mL)	Control (100) M (IQR)	IVIG+RBV (28) M (IQR) (Before treatment)	IVIG+RBV (28) M (IQR) (After treatment)	RBV (62) M (IQR) (Before treatment)	RBV (62) M (IQR) (After treatment)	χ2	*P*
IL-1β	3.00(2.95,3.53)^a^	15.30(8.48,42.70)^b^	3.25(3.00,3.50)^c,a^	14.95(8.50,42.35)^b^	3.45(3.20,3.90)^c,a^	113.926	< 0.001
IL-2	441.00(350.25,543.25)^a^	1017.00(726.00,1457.00)^b^	412.00(312.00,474.00)^c,a^	1014.50(725.00,1483.25)^b^	442.00(345.00,527.00)^c,a^	126.676	< 0.001
IL-6	4.05(3.20,4.70)^a^	16.00(6.78,82.60)^b^	4.20(3.60,5.10)^c,a^	15.95(4.78,85.50)^b^	4.70(3.70,5.70)^c,a^	72.654	< 0.001
IL-8	46.00(36.75,52.75)^a^	225.00(57.70,746.00)^b^	47.00(38.00,52.00)^c,a^	178.00(40.70,605.00)^b^	48.00(40.00,55.00)^c,a^	47.292	< 0.001
IL-10	5.10(3.68,6.25)^a^	7.82(6.28,10.20)^b^	4.50(3.60,6.40)^c,a^	8.55(6.41,14.85)^b^	4.10(2.80,6.25)^c,a^	61.261	< 0.001
IFN-γ	7.00(2.00,11.00)^a^	47.00(39.00,57.00)^b^	9.40(7.50,19.50)^c,a^	39.10(34.00,52.00)^b^	8.00(7.00,12.00)^c,a^	25.941	< 0.001
TNF-α	4.65(3.75,5.40)^a^	25.90(16.53,40.85)^b^	4.20(3.20,5.00)^c,a^	25.05(12.75,41.98)^b^	4.80(3.80,5.30)^c,a^	145.775	< 0.001

Abbreviations: *IVIG* intravenous gamma globulin intervention, *RBV* ribavirin, *M* median, *IQR* interquartile range, *IL* interleukin, *IFN* interferon, *TNF* tumor necrosis factor

a vs b, b vs c statistical significance

**Table 4:** Comparison of serum cytokines levels of healthy control, severe CV-A16 HFMD before and after treated with IVIG + RBV, and severe CV-A16 HFMD before and after treated with RBV only

Cytokine (pg/mL)	Control (100) M (IQR)	IVIG+RBV (19) M (IQR) (Before treatment)	IVIG+RBV (19) M (IQR) (After treatment)	RBV (125) M (IQR) (Before treatment)	RBV (125) M (IQR) (After treatment)	χ2	*P*
IL-1β	3.00(2.95,3.53)^a^	12.20(7.9199.68)^b^	3.15(3.00,3.55)^c,a^	16.80(8.79,71.00)^b^	3.30(3.00,3.60)^c,a^	126.232	< 0.001
IL-2	441.00(350.25,543.25)^a^	1372.50(924.50,1987.25)^b^	412.00(345.00,500.50)^c,a^	1085.50(837.50,1472.00)^b^	427.00(345.00,527.00)^c,a^	183.044	< 0.001
IL-6	4.05(3.20,4.70)^a^	18.20(7.44,33.30)^b^	4.40(3.60,4.95)^c,a^	11.50(5.06,90.20)^b^	4.20(2.78,5.30)^c,a^	86.937	< 0.001
IL-8	46.00(36.75,52.75)	158.00(11.80,216.00)	47.00(38.00,52.00)	63.10(21.60,571.00)	49.00(38.5,52.00)	5.638	0.228
IL-10	5.10(3.68,6.25)^a^	9.79(6.57,18.10)^b^	4.50(3.20,6.10)^c,a^	7.35(5.70,14.70)^b^	4.35(3.00,6.15)^c,a^	77.263	< 0.001
IFN-γ	7.00(1.60,11.00)^a^	47.00(38.00,51.50)^b^	7.50(5.00,11.50)^c,a^	60.20(41.00,75.40)^b^	8.00(5.30,12.00)^c,a^	15.329	0.004
TNF-α	4.65(3.75,5.40)^a^	25.80(16.40,41.40)^b^	4.20(3.70,5.20)^c,a^	19.05(13.90,52.18)^b^	4.70(3.80,5.50)^c,a^	192.151	< 0.001

Abbreviations: *IVIG* intravenous gamma globulin intervention, *RBV* ribavirin, *M* median, *IQR* interquartile range, *IL* interleukin, *IFN* interferon, *TNF* tumor necrosis factor

a vs b, b vs c statistical significance

### Management and outcomes of severe EV-A71 and CV-A16 HFMD

In order to prevent serious neurological and cardiopulmonary complications (aseptic meningitis, encephalitis, encephalomyelitis, acute flaccid paralysis, or autonomic nervous system dysregulation, pulmonary edema, pulmonary hemorrhage, and cardiorespiratory failure), antiviral treatments were performed either with RBV or IVIG in all hospitalized children. All 234 admitted subjects were treated with RBV, 47 children (47/234, 20.08%) with prolonged hyperthermia, and/ or neurological manifestations were treated with IVIG in addition to RBV (Table 1). The number of children treated with IVIG was significantly higher in EV-A71 (28/90, 31.11%) than CV-A16 (19/144, 13.19%) group (Table 1). As shown in Table 5, the parents of 31 children with prolonged hyperthermia, and/ or neurological manifestations refused to receive IVIG because of the high cost or safety concern. The duration of illness episodes of subjects treated with IVIG were significantly shorter than subjects without IVIG, including fever, vomiting, startle, myoclonic twitching, and length of stay (Table 5). None of the subject was under PICU care, and no fatal case identified. Finally, all of 234 admitted children were recovered and discharged without any complications.

**Table 5:** Comparison of clinical and laboratory outcomes of severe HFMD with prolonged hyperthermia, and/ or neurological manifestations treated with or without IVIG

Variable	IVIG (47) M (IQR)	Without IVIG^a^ (31) M (IQR)	Z	*P*
Fever	1 (1,1)	4 (4,4)	−9.971	< 0.001
Vomiting	2 (2,2)	3 (3,3)	−8.596	< 0.001
Startle	2 (2,2)	3 (3,4)	−9.711	< 0.001
Myoclonic twitching	2 (2,2)	4 (3.5,4)	−9.483	< 0.001
WBC (> 15 × 10^9^/L)	2 (2,2)	3 (3,3.5)	−9.729	< 0.001
CRP (> 40 mg/L)	2 (2,2)	4 (4,4)	−10.489	< 0.001
Length of stay (day)	4 (4,5)	5 (5,6)	−6.651	< 0.001

Abbreviations: *M* median, *IQR* interquartile range, *IVIG* intravenous gamma globulin intervention. ^a^Subjects with prolonged hyperthermia, and/ or neurological manifestations refused to receive IVIG because of the cost or safety issue

## Discussion

HFMD is a transmissible infectious disease caused by human EV that threats the health of children globally. A national surveillance system was established to monitor the epidemiology and aetiology of HFMD in China since 2008. It has been shown that EV-A71 and CV-A16 caused diseases were prevalent in China [15]. Epidemiological studies showed that HFMD had a seasonal circulation pattern of semi-annual outbreaks in May–July and September–October over the last few years in Shanghai and other cities in China [21–23]. In this study, we retrospective analyzed the records of clinical visit and admissions with probable HFMD in Xinhua Hospital from 2014 to 2016. The data showed that total 19,995 children with clinical findings suggestive of HFMD were brought forward for examination, and the major outbreaks appeared in May to September each year. Most of subjects were mild with only 2.87% (574/19,995) admitted after clinical assessment. EV-A71 and CV-A16 infections were confirmed by real-time RT-PCR among the admitted subjects, 90 children (90/574, 15.68%) were EV-A71 positive, and 144 children (144/574, 25.08%) were CV-A16 positive. EV-A71 and CV-A16 were mainly circulating in May to October following the outbreaks of probable HFMD pattern in Shanghai. EV-A71 presented the first peak of admissions from May to July, and CV-A16 appeared later from July to October. There were more males than females, and most of the admitted children were under 5 years. Similar to the previous studies [24, 25], the most common initial clinical symptoms of enrolled HFMD were fever and high WBC count. And laboratory test showed that CRP was elevated in most of hospitalized subjects. In addition, EV-A71 was reported as a neurotropic virus associated with neurological complications. Our data also showed that the incidences of neurological symptoms, such as myoclonic twitching and startle were higher in EV-A71 than CV-A16 cases. We also noted that the number of admitted severe disease children was decreased from 2014 to 2016 in Xinhua Hospital.

Inflammatory cytokines are the molecular proteins of the host immune response during viral infection, which are postulated that impact the pathogenicity and severity of HFMD [26]. Viral infection activates cytokine networks and increases levels of various cytokines in blood [26]. The upregulated cytokines levels may be associated with clinical presentations of HFMD. It was reported that several cytokines play roles in the regulation of inflammation and fever, such as IL-1β, IL-6 and TNF-α [26]. In addition, IFN-γ, IL-1β, IL-2, IL-6, IL-8 and IL-10 have been demonstrated to be involved in the proliferation of immune cells, including T and B lymphocytes [27]. It has been demonstrated that upregulation of inflammatory cytokines may cause neurological complications, cardiopulmonary collapse and higher fatality following EV-A71 infection in children [26]. An early study [28] showed that even mild HFMD patients without neurological complications had elevated serum levels of inflammatory cytokines. Another study [29] found that cytokine profiles were varied between the patients with mild and severe EV17-related HFMD, which may indicate prognosis and strain virulence. In this study, the serum levels of IL-1β, IL-2, IL-6, IL-8, IL-10, IFN-γ, and TNF-α were measured by ELISA in admitted EV-A71 and CV-A16 HFMD subjects. The data showed that cytokine levels of IL-1β, IL-2, IL-6, IL-8, IFN-γ, and TNF-α were significantly elevated in EV-A71 and CA16 subjects compared with either mild HFMD or healthy control group. However, no significantly difference between EV-A71 and CV-A16 group in most of those cytokines, except the IFN-γ level was higher in CV-A16 than EV-A71 group. Among subjects with severe HFMD there was no statistical difference in their cytokine levels irrespective of whether they were given IVIG, suggesting that cytokine levels may not be an indicator for severe HFMD due to EV-A71 and CV-A16. The serum cytokines levels of all children were back to normal after treatment with IVIG or RBV before discharged (data not shown).

Although there is no approved specific antiviral treatment for HFMD, all hospitalized children were treated with either RBV or IVIG based on the clinical symptoms in this study. A prospective, multicenter, randomized, double-blind and placebo-controlled trial included 300 HFMD outpatients showed that RBV aerosol had better clinical efficacy on viral exclusion than placebo group [30]. Another randomized, double-blind, placebo-controlled trial included 119 HFMD patient showed that the RBV aerosol group had a significantly higher overall negative conversion rate of EV than the control group [31]. Furthermore, RBV was also used to treat severe HFMD patients needed intensive care [32]. IVIG has been suggested to treat severe EV infections based on evidence of a possible significant benefit through the reduction of the associated central nervous system (CNS) inflammatory response [7]. Studies [33, 34] showed that administration of high-dose IVIG achieved good anecdotal outcomes in EV-A71 outbreaks for severe HFMD subjects. Our previous study [17] also showed changes in outcome by early use of IVIG in outbreaks of EV-A71 infection during 2010 to 2012 in Shanghai. In this study, more improved outcomes with no fatal case may partly be due to using of IVIG, particularly in EV-A71 HFMD cases. More EV-A71 infected children were suggested to receive IVIG because of neurological symptoms. However, there are still no well-designed prospective randomized trials to investigate the effects and benefits of IVIG in treating severe HFMD. The systematic use of IVIG is still controversial by considering that IVIG may not contain adequate quantities of antibodies to neutralize the large number of EV serotypes and subtypes [7]. Our current study further supports the anecdotal benefit of IVIG in shorten duration of the illness episodes if given early in severe HFMD. However, many parents of the children refused to use IVIG because of the high cost and safety concern of human blood product. Effective vaccines remain the best way to overcome HFMD. For example, clinical trials showed that EV-A71 C4a vaccines developed in China had been proved to be immunogenic, safe and capable of conferring protection in most of the vaccinated individuals [35, 36]. Therefore, China Food and Drug Administration (CFDA) had licensed EV-A71 C4a vaccines for use in humans since 2016. Vaccines for other EV serotypes are needed to be developed to prevent the outbreaks of HFMD in the future.

There are several limitations in the present study. Firstly, given that Xinhua Hospital is not the only hospital serving children in the Shanghai region, this cohort may not be representative with respect to regional factors. Secondly, selection bias may be present we only selected confirmed EV-A71 and CV-A16 infections of the admitted severe HFMD cases, which only account for about 40% of total hospitalized children. Subjects caused by other EV should be confirmed and included for study in the future. Thirdly, those subjects who received IVIG were not randomized, resulting in biases in analysis of outcomes of subjects treat with or without IVIG.

## Conclusions

A total of 19,995 children visited Xinhua Hospital clinic service with probable HFMD during 2014–2016. The admission rate was 2.87%. EV-A71 and CV-A16 were major causes of admitted severe HFMD. IVIG appeared to be beneficial in shortening the duration of illness episodes of severe HFMD. Further well-designed studies are needed to investigate the effect of IVIG in treatment of severe HFMD.

## Abbreviations

ALT: Alanine aminotransferase
BS: Blood sugar
CFDA: China food and drug administration
CKMB: Creatinekinase MB
CNS: Central nervous system
CRP: C reactive protein
CSF: Cerebral spinal fluid
CV: coxsackievirus
ELISA: Enzyme-linked immunosorbent assay
EVs: enteroviruses
HFMD: Hand, foot and mouth disease
IFN-γ: Interferon-γ
IL: Interleukin
IQR: Interquartile range
IVIG: Intravenous gamma globulin
PICU: Pediatric intensive care unit
RBV: Ribavirin
TNF-α: Tumor necrosis factor-α
WBC: White blood cells
